# Supplementary material for: Maternal occupational risk factors and preterm birth: Protocol for a systematic review and meta-analysis
Source: PLoS One. 2023 Jul 11;18(7):e0283752. doi: 10.1371/journal.pone.0283752 (PMC10335685; doi:10.1371/journal.pone.0283752)
Supplement: S2 File — (DOCX) [file pone.0283752.s002.docx]

**Table 1: Draft of search strategy to be used using Medline electronic database.**

| **Number** | **Search terms** |
| --- | --- |
| 1 | Occupational Exposure/ |
| 2 | Lifting/ or Workload/ or employment/ or standing position/ |
| 3 | Shift Work Schedule/ or Work Schedule Tolerance/ |
| 4 | “Occupational Exposure*”.mp. |
| 5 | (occupational activit* or workload).mp. |
| 6 | ((activ* adj2 (intens* or physical* or vigor*)) and (work* or occupation*)).mp. |
| 7 | (heavy exertion* or heavy lifting or heavy work*).mp. |
| 8 | (((demand* or drain* or exhaust* or fatigu*) adj2 physical*) and (work* or occupation*)).mp. |
| 9 | (prolonged adj5 (standing or walking or bending or upright)).mp. |
| 10 | (long work* hour* or long work* day*).mp. |
| 11 | ''whole-body vibration''.mp. |
| 12 | working time.mp. |
| 13 | (shift work or afternoon shift or evening shift or morning shift or night shift or rotating shift or shift schedule).mp. |
| 14 | 1 or 2 or 3 or 4 or 5 or 6 or 7 or 8 or 9 or 10 or 11 or 12 or 13 |
| 15 | Premature Birth/ |
| 16 | infant, premature/ or infant, extremely premature/ |
| 17 | Obstetric Labor, Premature/ |
| 18 | ((preterm or pre-term or premature) adj (birth* or infan* or babby or babies or neonat* or labo?r or deliver*)).mp. |
| 19 | extreme* prematur*.mp. |
| 20 | 15 or 16 or 17 or 18 or 19 |
| 21 | 14 and 20 |
| 22 | pregnancy outcome/ or pregnancy complication/ |
| 23 | (“adverse pregnancy outcome” or “adverse birth outcome”).mp. |
| 24 | 22 or 23 |
| 25 | 14 and 24 |
| 26 | 21 or 25 |
| **27** | **Limit to year="2000 -Current"** |

**Table 2: Draft of search strategy to be used using Embase electronic database.**

| **Number** | **Search terms** |
| --- | --- |
| 1 | Occupational Exposure/ or whole-body vibration/ |
| 2 | Lifting/ or Workload/ or standing/ or erect posture/ |
| 3 | Shift Work Schedule/ or Work Schedule Tolerance/ or Working time/ or night work/ |
| 4 | “Occupational Exposure”.mp. |
| 5 | (occupational activit* or workload).mp. |
| 6 | ((activ* adj2 (intens* or physical* or vigor*)) and (work* or occupation*)).mp. |
| 7 | (heavy exertion* or heavy lifting or heavy work*).mp. |
| 8 | (((demand* or drain* or exhaust* or fatigu*) adj2 physical*) and (work* or occupation*)).mp. |
| 9 | (prolonged adj5 (standing or walking or bending or upright)).mp. |
| 10 | (long work* hour* or long work* day*).mp. |
| 11 | ''whole-body vibration''.mp. |
| 12 | working time.mp. |
| 13 | (shift work or afternoon shift or evening shift or morning shift or night shift or rotating shift or shift schedule).mp. |
| 14 | 1 or 2 or 3 or 4 or 5 or 6 or 7 or 8 or 9 or 10 or 11 or 12 or 13 |
| 15 | Premature Birth/ |
| 16 | infant, premature/ or infant, extremely premature/ |
| 17 | Obstetric Labor, Premature/ |
| 18 | ((preterm or pre-term or premature) adj (birth* or infan* or babby or babies or neonat* or labo?r or deliver*)).mp. |
| 19 | extreme* prematur*.mp. |
| 20 | 15 or 16 or 17 or 18 or 19 |
| 21 | 14 and 20 |
| 22 | pregnancy outcome/ or birth outcome/ |
| 23 | ("adverse pregnancy outcome" or "adverse birth outcome").mp |
| 24 | 22 or 23 |
| 25 | 14 and 24 |
| 26 | 21 or 25 |
| **27** | **Limit to year="2000 -Current"** |

**Table 3: Draft of search strategy to be used using Emcare electronic database.**

| **Number** | **Search terms** |
| --- | --- |
| 1 | Occupational Exposure/ or Occupational hazard/ or Occupational health/ or whole-body vibration/ |
| 2 | Lifting/ or Workload/ or standing/ or erect posture/ or Employment/ |
| 3 | Shift Work Schedule/ or Work Schedule Tolerance/ or Working time/ or night work/ |
| 4 | “Occupational Exposure”.mp. |
| 5 | (occupational activit* or workload).mp. |
| 6 | ((activ* adj2 (intens* or physical* or vigor*)) and (work* or occupation*)).mp. |
| 7 | (heavy exertion* or heavy lifting or heavy work*).mp. |
| 8 | (((demand* or drain* or exhaust* or fatigu*) adj2 physical*) and (work* or occupation*)).mp. |
| 9 | (prolonged adj5 (standing or walking or bending or upright)).mp. |
| 10 | (long work* hour* or long work* day*).mp. |
| 11 | ''whole-body vibration''.mp. |
| 12 | working time.mp. |
| 13 | (shift work or afternoon shift or evening shift or morning shift or night shift or rotating shift or shift schedule).mp. |
| 14 | 1 or 2 or 3 or 4 or 5 or 6 or 7 or 8 or 9 or 10 or 11 or 12 or 13 |
| 15 | Premature Birth/ |
| 16 | infant, premature/ or infant, extremely premature/ |
| 17 | Obstetric Labor, Premature/ |
| 18 | ((preterm or pre-term or premature) adj (birth* or infan* or babby or babies or neonat* or labo?r or deliver*)).mp. |
| 19 | extreme* prematur*.mp. |
| 20 | 15 or 16 or 17 or 18 or 19 |
| 21 | 14 and 20 |
| 22 | pregnancy outcome/ or pegnancy complication/ |
| 23 | ((pregnanc* or birth*) adj (outcom* or complicatio*)).mp. |
| 24 | 22 or 23 |
| 25 | 14 and 24 |
| 26 | 21 or 25 |
| **27** | **Limit to year="2000 -Current"** |

**Table 4: Draft of search strategy to be used using CINHAL electronic database.**

| **Number** | **Search terms** |
| --- | --- |
| 1 | (MH "Occupational Exposure") |
| 2 | (MH "Occupational Hazards") |
| 3 | (MH "Occupational Health") |
| 4 | (MH "Lifting") |
| 5 | (MH "Workload") |
| 6 | (MH "Standing") |
| 7 | (MH "Employment") |
| 8 | (MH "Shiftwork") |
| 9 | "Occupational Exposure" |
| 10 | ("occupational activit*" or workload) |
| 11 | ((activ* W2 (intens* or physical* or vigor*)) and (work* or occupation*)) |
| 12 | ("heavy exertion*" or "heavy lifting" or "heavy work*") |
| 13 | (((demand* or drain* or exhaust* or fatigu*) W2 physical*) and (work* or occupation*)) |
| 14 | (prolonged W5 (standing or walking or bending or upright)) |
| 15 | ("long work*" hour* or "long work*" day*) |
| 16 | ''whole-body vibration'' |
| 17 | "working time" |
| 18 | ("shift work" or "afternoon shift" or "evening shift" or "morning shift" or "night shift" or "rotating shift" or "shift schedule") |
| 19 | S1 OR S2 OR S3 OR S4 OR S5 OR S6 OR S7 OR S8 OR S9 OR S10 OR S11 OR S12 OR S13 OR S14 OR S15 OR S16 OR S17 OR S18 |
| 20 | (MH "Childbirth, Premature") |
| 21 | (MH "Infant, Premature") |
| 22 | (MH "Labor, Premature") |
| 23 | ((preterm or pre-term or premature) W1(birth* or infan* or babby or babies or neonat* or labo?r or deliver*)) |
| 24 | extreme* prematur* |
| 25 | S20 OR S21 OR S22 OR S23 OR S24 |
| 26 | S19 AND S25 |
| 27 | (MH "Pregnancy Outcomes") |
| 28 | MH "Pregnancy Complications") |
| 29 | ((pregnanc* or birth*) W1 (outcom* or complicatio*)) |
| 30 | S27 OR S28 OR S29 |
| 31 | S19 AND S30 |
| 32 | S26 OR S31 |
| **33** | **Limit to year="2000 -Current"** |

**Table 5: Draft of search strategy to be used using Scopus electronic database.**

| **Number** | **Search terms** |
| --- | --- |
| 1 | “Occupational Exposure” |
| 2 | (“occupational activit*” or workload) |
| 3 | ((activ* W/2 (intens* or physical* or vigor*)) and (work* or occupation*)) |
| 4 | (“heavy exertion*” or “heavy lifting” or “heavy work*”) |
| 5 | (((demand* or drain* or exhaust* or fatigu*) W/2 physical*) and (work* or occupation*)) |
| 6 | (prolonged W/2 (standing or walking or bending or upright)) |
| 7 | (“long work*” hour* or “long work*” day*) |
| 8 | “whole-body vibration” |
| 8 | “working time” |
| 9 | (“shift work” or “afternoon shift” or “evening shift” or “morning shift” or “night shift” or “rotating shift” or “shift schedule”) |
| 10 | 1 or 2 or 3 or 4 or 5 or 6 or 7 or 8 or 9 or 10 or 11 or 12 or 13 |
| 11 | ((preterm or “pre-term” or premature) W/2 (birth* or infan* or babby or babies or neonat* or labo?r or deliver*)) |
| 12 | extreme* prematur* |
| 13 | 11 or 112 |
| 14 | 10 and 13 |
| **19** | **Limit to year="2000 -Current"** |

**Table 6: Draft of search strategy to be used using Web of Science electronic database.**

| **Number** | **Search terms** |
| --- | --- |
| 1 | “Occupational Exposure” |
| 2 | (“occupational activit*” or workload) |
| 3 | ((activ* NEAR/2 (intens* or physical* or vigor*)) and (work* or occupation*)) |
| 4 | (“heavy exertion*” or “heavy lifting” or “heavy work*”) |
| 5 | (((demand* or drain* or exhaust* or fatigu*) NEAR/2 physical*) and (work* or occupation*)) |
| 6 | (prolonged NEAR/2 (standing or walking or bending or upright)) |
| 7 | (“long work*” hour* or “long work*” day*) |
| 8 | “whole-body vibration” |
| 8 | “working time” |
| 9 | (“shift work” or “afternoon shift” or “evening shift” or “morning shift” or “night shift” or “rotating shift” or “shift schedule”) |
| 10 | 1 or 2 or 3 or 4 or 5 or 6 or 7 or 8 or 9 or 10 |
| 11 | ((preterm or “pre-term” or premature) NEAR/2 (birth* or infan* or babby or babies or neonat* or labo?r or deliver*)) |
| 12 | extreme* prematur* |
| 13 | “adverse pregnancy outcome” |
| 13 | 11 or 112 or 13 |
| 14 | 10 and 13 |
| **19** | **Limit to year="2000 -Current"** |
