# Supplementary material for: Maternal occupational risk factors and preterm birth: Protocol for a systematic review and meta-analysis
Source: PLoS One. 2023 Jul 11;18(7):e0283752. doi: 10.1371/journal.pone.0283752 (PMC10335685; doi:10.1371/journal.pone.0283752)
Supplement: Attachment — Submitted filename: Authors response.docx [file pone.0283752.s003.docx]

**Authors response**

**Maternal occupational risk factors and Preterm birth: Protocol for a systematic review and meta-analysis**

**Corresponding Author; Haimanot Abebe** [**Adane: haimanot.adane@monash.edu**](mailto:Adane:%20haimanot.adane@monash.edu)

**Authors**

**Haimanot Abebe** [**Adane: Haimanot.Adane@monash.edu**](mailto:Adane:%20Haimanot.Adane@monash.edu)

**Ross Iles:** [**ross.iles@monash.edu**](mailto:ross.iles@monash.edu)

**Jacqueline A. Boyle:** [**jacqueline.boyle@monash.edu**](mailto:jacqueline.boyle@monash.edu)

**Alex Collie:** [**Alex.Collie@monash.edu**](mailto:Alex.Collie@monash.edu)

**Manuscript ID:** PONE-D-22-29810

**Journal:** PLOS ONE

**Article type: Study Protocol (Systematic review and meta-analysis)**

**To editors and reviewers**

First of all, the authors would like to express their gratitude to the editors of the “PLOS ONE” journal editor and the respected reviewers for reviewing our manuscript and offering valuable suggestions to enhance its scientific merit. We have updated the manuscript with corrections in response to the comments made. As a result, all comments have been accepted and integrated into the revised manuscript.

**A point-by-point response to Editor**

**Editor:** Consider reviewing the inclusion of manuscripts exclusively published in English in the protocol, as today with tools such as Google Translate and others, language limitations in the search strategy for a systematic review are not justified, as they facilitate the translation and understanding of the content of articles published in other languages. A search only for manuscripts published in English causes an important bias that limits the external validity of the systematic review, as well as compromises its results

**Authors:** While the exclusion of non-English language articles may lead to bias and miss important articles, some scholars have argued that the exclusion of non-English articles has a limited impact on the finding and overall conclusion of the review (<https://doi.org/10.1016/j.jclinepi.2019.10.011>). From a practical perspective excluding non-English language articles during the search stage of a review risks the exclusion of relevant non-English language articles, where language values have been incorrectly defined or are missing. Excluding non-English language articles during the eligibility assessment stage instead allows the reason for ineligibility to be recorded, providing greater transparency about the number of articles excluded on this basis. We will exclude non-English language at the eligibility assessment stage due to the practical barriers such as the high cost and time commitment associated with translating articles. We have considered the limitations of translation software (e.g Google Translate): Based on our experience, and scholarly point of view google translate often produces translations that contain significant grammatical errors and do not have a system to correct translation errors [https://doi.org/10.1186/2046-4053-2-97]. This may lead to missing or misinterpreting key evidence, which may limit the generalisability of findings.

**Editor:** Finally, why did you decide to include only manuscripts published after the year 2000 in your search strategy? Are there no studies relating maternal occupational risk factors and preterm birth published before 2000? If they exist, the authors need to review this criterion.

**Authors:** The reason for deciding to include only articles published after the year 2000 is that, over the years, not only has the proportion of women in the workforce changed, but also the working conditions for pregnant women. Recently it is more common to have modifications of working conditions during pregnancy, paid maternity leave, or health benefits by law. To provide a contemporary answer to whether or not nowadays specific physical activities or working conditions exert an influence on preterm birth, we only seek recent studies for this systematic review using more recent studies will give a better reflection of today’s risk of preterm birth. By limiting studies to published in 2000 or more recent, we will be reviewing more than 20 years of research while ensuring the findings reflect contemporary working conditions.

**Editor**: Another recommendation is that the authors add keywords related to the types of study in table 1, such as prospective cohort, retrospective cohort, case-control and cross-sectional studies.

**Authors:** The goal of systematic review searches is to identify all relevant studies on a topic. Systematic review searches are therefore typically quite extensive. However, it may be necessary to strike a balance between the sensitivity and precision of our search. Increasing the comprehensiveness of a search will reduce its precision and will retrieve more non-relevant results. Thus, considering your suggestion we follow the standard search strategy technique (PICO).

**A point-by-point response to (Reviewer # 1)**

**Reviewer 1:** (Introduction) I think that a systematic review of this topic is important. In contrast, could you explain in the Introduction why a protocol for the systematic review needs to be published? I think that the authors should submit a manuscript after conducting the research.

**Authors:** The importance of publishing our protocol for the systematic review appears **Line 111-114**). We do intend to submit a manuscript detailing the review findings.

**Reviewer 1:** (Introduction) Weren’t there any systematic reviews that investigated an association between preterm birth and occupational physical risk in the past?

**Authors:** While there are previous reviews exist, the reasons we are conducting this SLR and Meta-analysis are briefly described in **Lines 93-100**. In more detail:

1- While the evidence from previous reviews is useful, their authors have reported conflicting or weak evidence and as such have concluded that it is challenging to provide explicit recommendations for clinical practice or policy.
2- A number of prior reviews have not utilised rigorous methodological standards for reporting on study quality
3- None of the reviews examined the impacts of whole-body vibration on preterm birth, and nor have they sought to differentiate between medically indicated or spontaneous preterm birth
4- Further, the included evidence in most reviews reflects working conditions of the 1960’s and 2000’s. In many occupations and nation, working conditions have changed dramatically throughout the early 21^st^ century and thus the nature, prevalence and impacts of occupational physical health risks has also changed.

**Reviewer 1:** (Meta-analysis section in Methods) It is written hat “a pooled odds ratio from ~ risk ratio”, how do you obtain a pooled odds ratio from risk ratio? Using risk ratio as a odds ratio for some studies might not be a good idea.

**Authors:** We intend to transform risk ratios into odd ratios by using the formula; RR = OR / (1 – p + (p x OR)), where p is the risk in the control group.

**A point-by-point response to (Reviewer # 2)**

**Reviewer 2:** This manuscript by Adane et al. is useful for public health reading, especially in the area of maternal and fetal health. Minor comments Consistency with "preterm" or "pre-term"

**Authors:** This is now consistent throughout the manuscript.

**Reviewer 2:** Figure 1 has (n=) with no explanation for its meaning

**Authors:** We have made corrections. (See; **Line 174**)

**Reviewer 2:** Inclusion criteria: Both singleton and twin pregnancies, nulliparous and multiparous included? Please clarify

**Authors:** We have clarified in the revised manuscript that we will include a singleton pregnancy and both nulliparous and multiparous women. (See; **Line 125**) Since over 60% of twin and nearly all higher-order multiples are premature (born before 37 weeks) and intended to control the confounding effect of multiple births we only include a singleton pregnancy.
